# Different currencies for calculating resource phenology result in opposite inferences about trophic mismatches

**DOI:** 10.1098/rspb.2023.1785

**Published:** 2024-03-27

**Authors:** Tom S. L. Versluijs, Mikhail K. Zhemchuzhnikov, Dmitry Kutcherov, Tomas Roslin, Niels Martin Schmidt, Jan A. van Gils, Jeroen Reneerkens

**Affiliations:** ^1^ NIOZ Royal Netherlands Institute for Sea Research, PO Box 59, 1790 AB Den Burg, The Netherlands; ^2^ University of Groningen, PO Box 72, 9700 AB Groningen, The Netherlands; ^3^ Department of Biological Sciences, University of Arkansas, Fayetteville, AR 72701, USA; ^4^ Department of Ecology, Swedish University of Agricultural Sciences (SLU), Ulls väg 18B, 75651 Uppsala, Sweden; ^5^ Organismal and Evolutionary Biology Research Programme, Faculty of Biological and Environmental Sciences, University of Helsinki, PO Box 65, Viikinkaari 1, Helsinki 00014, Finland; ^6^ Department of Ecoscience, Aarhus University, 4000 Roskilde, Denmark; ^7^ Arctic Research Centre, Aarhus University, 8000 Aarhus, Denmark; ^8^ Sovon Dutch Centre for Field Ornithology, PO Box 6521, 6503 GA, Nijmegen, The Netherlands

**Keywords:** allometry, ecological interactions, phenological mismatch, phenology, terrestrial ecology, trophic interactions

## Abstract

Shifts in phenology are among the key responses of organisms to climate change. When rates of phenological change differ between interacting species they may result in phenological asynchrony. Studies have found conflicting patterns concerning the direction and magnitude of changes in synchrony, which have been attributed to biological factors. A hitherto overlooked additional explanation are differences in the currency used to quantify resource phenology, such as abundance and biomass. Studying an insectivorous bird (the sanderling) and its prey, we show that the median date of cumulative arthropod biomass occurred, on average, 6.9 days after the median date of cumulative arthropod abundance. In some years this difference could be as large as 21 days. For 23 years, hatch dates of sanderlings became less synchronized with the median date of arthropod abundance, but more synchronized with the median date of arthropod biomass. The currency-specific trends can be explained by our finding that mean biomass per arthropod specimen increased with date. Using a conceptual simulation, we show that estimated rates of phenological change for abundance and biomass can differ depending on temporal shifts in the size distribution of resources. We conclude that studies of trophic mismatch based on different currencies for resource phenology can be incompatible with each other.

## 1. Introduction

Climate change is restructuring communities across the globe. Among the key responses of organisms to climate-driven changes in abiotic conditions are changes in phenology (i.e. changes in season-related life cycle events) [1,2]. Several studies have indicated that the magnitude and direction of phenological change can vary between trophic levels [3–5], potentially resulting in a decoupling of the phenological relations between interacting species and ultimately affecting demographic parameters [6,7].

Phenological mismatches between trophic levels have been studied across a wide variety of ecosystems and taxonomic groups [4,8,9]. This includes antagonistic interactions between predator and prey [10,11], herbivores and plants [5,12], and mutualistic interactions between plants and pollinators [13,14]. Several studies have attempted to integrate results of studies on mismatches in consumer-resource interactions across functional groups, ecosystems and/or biomes [3,15,16]. However, results are often partially conflicting regarding the direction and magnitude of changes in synchrony [9,15,17], whether observed asynchrony scales with covariates such as latitude or the degree of warming [15,16], or whether observed asynchrony correlates with changes in demographic parameters of the consumer [9,18,19].

The lack of consistent results in large-scale (meta-)analyses can, to a large extent, be attributed to biological factors. Variability in trends of (a)synchrony between phenological events [20] might arise due to differences in the ecological conditions and different local trends in the climate to which interacting species are exposed [21]. Variability might also arise due to species-specific differences in life histories [22,23], in the phenological cues to which different trophic levels respond [12,17], in their phenological sensitivity to the same cue, or in the time window during which they respond to this cue [8,24,25]. Furthermore, there might be additional selective pressures to which consumers are exposed, making asynchrony with their resource in fact adaptive [18,26].

However, discrepancies in the results of large-scale meta-analyses might additionally arise due to differences in approaches among selected studies. First, the duration and period of time series in included studies varies considerably, which can impact the degree and direction of changes in (a)synchrony detected [3]. Second, studies vary in the level at which phenological data are collected, ranging from data on individuals, through populations to communities [27]. Third, different indicators are used to approximate resource phenology [16]. For instance, measurements of chlorophyll *a* are frequently used as an indicator for phytoplankton biomass [28], even though temporal dynamics in chlorophyll quantities and plankton biomass can differ substantially [29]. Fourth, changes in consumer phenology have generally been expressed relative to changes in the phenology of a comparative ‘yardstick’, such as the phenology of the consumer's resource [17]. Many different metrics have been used between studies to define this yardstick, including the timing of peak abundance, the moment of initiation, or the moment when a cumulative threshold is passed [30,31]. This variability in metrics hampers comparisons between studies, as this will not only impact estimates of rates of phenological change [32,33], but also their precision [34], ultimately impacting inferences regarding the drivers of observed phenological change [30,35]. Fifth, different models have been used to quantify the degree of mismatch between a consumer and its resource [23,36], whereas the choice of a model can influence estimates of mismatch and thereby affect any downstream inference regarding their impact on the vital rates of consumers [37,38].

An additional (often overlooked) source of methodological variation is the actual unit of the measurements (hereafter ‘currency’) used to calculate a phenological metric. Across systems, the most frequently used currencies to quantify resource phenology are abundance (i.e. numbers or densities) and biomass. These currencies are often used interchangeably, with some studies on trophic mismatches between arthropods and birds quantifying arthropod phenology based on arthropod abundance [39,40], while others use estimates of arthropod biomass [41–43]. Similarly, phenological studies of phytoplankton sometimes use abundance [4,44], and other times use (inferred) biomass [32,33] to quantify phenology.

Importantly, temporal trends in biomass or abundance are not necessarily correlated, because trends in biomass might be dominated by large-bodied taxa that are not numerically abundant, while trends in abundance are generally dominated by small-bodied and numerically abundant taxa [45,46]. When taxa of different sizes vary in their phenology [44,47], or in their phenological shifts [44,48], the use of abundance or biomass might result in different phenological estimates or rates of phenological change, respectively. Despite the potential impact of different currencies on estimates of phenology, its consequences have, to the best of our knowledge, not been directly assessed in the context of trophic mismatches.

In this study, we assess how our perception of a trophic mismatch is impacted by the currencies used to quantify resource phenology. As a case example, we focus on a terrestrial antagonistic interaction occurring between insectivorous birds and their arthropod prey. We find that our ecological inference will depend entirely on the currency chosen. This concerns both the extent and direction of asynchrony between consumer and resource, and the magnitude of estimated rates of phenological change. These findings, we argue, reveal the fundamental importance of the methodological choices made in studies of trophic mismatches, and suggests that studies of trophic mismatch based on different currencies should be regarded as fundamentally incompatible with each other.

## 2. Methods

### (a) Estimates of phenological synchrony between trophic levels based on arthropod biomass versus arthropod abundance

To evaluate how our choice of currency for prey phenology will affect our perception of a trophic mismatch, we introduce a model system: the breeding phenology of an arctic shorebird—the sanderling (*Calidris alba*)—and the phenology of its arthropod prey. We derive measures of arthropod phenology in terms of abundance and biomass (with the latter inferred using allometric length-biomass regressions). For this purpose we analysed 23 years of arthropod and bird data collected at Zackenberg in high arctic Greenland [39,49,50]. In addition, we use a simple conceptual simulation to assess how rates of phenological change are affected by the choice of currency for prey phenology.

#### (i) Arthropod data

Since 1996 arthropod communities have been sampled throughout the snow-free season at Zackenberg (74°28′ N, 20°34′ W). This monitoring took place at seven plots situated in wet fens and dry heaths [49,51]. Each plot measured 10 × 20 m² and contained eight yellow pitfall traps until 2007 and four pitfall traps from 2007 onwards [49]. Sampling at each plot took place at near-weekly intervals from snowmelt until late August or late September. All specimens in our analysis were identified to family level. We only included specimens belonging to the orders Araneae, Coleoptera, Diptera, Hemiptera, Hymenoptera and Lepidoptera as these comprise the main part of the sanderling diet [52]. Thus, we excluded Acari and Collembola, which are numerically the most abundant taxonomic groups in Zackenberg [51], but make a (very) limited contribution to overall biomass. We excluded one plot that was not operational between 1999 and 2018 [49]. We truncated our analysis to a fixed time window from day of year 157 (5–6 June) to 238 (25–26 August) to prevent biases due to differences in duration of the trapping period among years. We excluded year 2018, when excess snow cover resulted in only one sanderling nest being found [53], and year 2020 when, due to the COVID pandemic, arthropod sampling only started 13 days after the start of the selected fixed trapping window.

#### (ii) Arthropod abundance to biomass

To calculate annual seasonal trends in arthropod biomass at Zackenberg we first calculated the average number of specimens caught per taxonomic group per day per trap. This we did for each day of the time window during which a trap was active. These data only comprise counts of arthropods but do not contain measurements of the length or weight of individual specimens. Therefore, to calculate trends in biomass we first assigned a length to each specimen by sampling from an additional dataset containing taxon-specific and life-stage-specific (i.e. larvae versus adult) length distributions (electronic supplementary material, appendix S1 and section S1). Once a length was assigned to each specimen, we calculated each specimen's biomass using taxon-specific length–biomass regressions [54]. For each year, we then computed daily estimates of total arthropod biomass by summing daily biomass estimates over all taxonomic groups and averaging them across all active traps.

#### (iii) Arthropod phenology

To quantify arthropod phenology, we used linear interpolation to calculate (i) the date when 50% of cumulative abundance was reached (hereafter ‘median date of arthropod abundance’), and (ii) the date when 50% of cumulative biomass was reached (hereafter ‘median date of arthropod biomass’). We note that differences in phenological patterns of arthropod biomass versus arthropod abundance will occur mainly as a function of seasonal changes in individual-level body mass. Thus, to characterize the latter, we also fitted a generalized additive model [55] using cubic splines to the raw biomass data for all specimens throughout the season, both for all years combined and separately for each year.

#### (iv) Sanderling hatch dates

We searched for sanderling nests on foot in Zackenberg in June and July each year from 1996 to 2019 [39]. For each nest found during the incubation stage (*n* = 441), we estimated hatch dates by floating two eggs in warm water and assessing the flotation angle and height [56,57]. It was shown in Zackenberg that egg flotation did not affect egg hatchability in sanderlings and other shorebirds [57]. We then revisited nests approximately 2–3 days before the expected hatch date to check for signs of hatching to determine the exact hatch date [39,58]. We analysed estimated hatch dates such that unsuccessful nests (i.e. without an actual hatch date) could also be included in our analysis. We did not exclude assumed replacement clutches of sanderlings [58] because they could usually not be identified as such with certainty. Within a day after hatch, the (typically four) chicks leave the nest scrape located on the tundra surface, in search of ground-dwelling arthropods guided by one of the parents. We also estimated hatch dates of encountered families (*n* = 165), based on a logistic growth function, following [39], that was fitted to chick body mass growth data collected at Zackenberg from 2003 to 2017 (*n* = 460 weight measurements of 357 chicks of known age). We then calculated the median hatch date for each year for which we also estimated the median date of arthropod emergence.

#### (v) Phenological synchrony between trophic levels

To assess how different currencies for arthropod phenology can affect estimates of phenological (a)synchrony, we subtracted (i) the median date of arthropod abundance and (ii) the median date of arthropod biomass from the median hatch date of sanderling chicks for each year. We then used linear regression models to test for time trends in both estimates of synchrony over the study period. For all estimated parameters we obtained 95% quantile confidence intervals using non-parametric (case) bootstrapping with 10 000 bootstrap samples [59,60]. The synchrony between consumer and resource has frequently been quantified by calculating the temporal difference between their phenological peaks [7,11,15]. Although more advanced models also take the architecture of the resource peak into account [21,36], such models do not necessarily outperform simple ‘peak-date’ models [37]. We stress that our definition of synchrony only is a quantification of the degree of alignment of the median dates for consumer and resource. This does not imply that we associate cases of asynchrony between sanderlings and the median date in arthropod abundance or biomass with (negative) fitness consequences.

### (b) A conceptual simulation to assess the effect of different currencies on rates of phenological change

We constructed a simple conceptual simulation to illustrate how rates of phenological change can be affected by the choice of currency for prey phenology. The assumed distributions, parameter values and hypothetical scenarios in this simulation are qualitatively based on empirical studies on arctic arthropods (this study), tephritid flies and phytoplankton (see below) [61]. The exact parameter values were chosen such that differences in rates of phenological change between currencies are most visually distinct.

#### (i) From abundance to biomass for a single year

To illustrate the concept of how patterns in abundance translate to biomass, we first simulated a single year of abundance data. We modelled the phenological distribution of abundance by assuming a symmetrical Gaussian distribution. We then computed biomass from abundance by multiplying the distribution of abundance (i.e. number of specimens per day) with the relationship between the average biomass per specimen and date (i.e. biomass per specimen per day). To illustrate this concept, we assumed that the latter relationship was linear with a positive slope, indicating that smaller specimens have an earlier phenology than larger specimens. We then multiplied the phenological distribution of abundance with this linear relationship to obtain the phenological distribution of biomass. Subsequently, we repeated these steps with a skewed phenological distribution of abundance to assess the impact of a non-symmetrical distribution on phenological estimates for abundance and biomass.

#### (ii) Rates of phenological change for abundance and biomass

To illustrate how differences in the rates of phenological change between abundance and biomass can arise, we first modelled a single phenological distribution of abundance based on a symmetrical Gaussian distribution. The phenology of this distribution was then advanced linearly by a fixed number of days per year, for five consecutive years (i.e. assuming a constant rate of phenological change for abundance). To translate these phenological shifts in abundance to shifts in biomass, we multiplied each annual distribution in abundance with a (year-specific) linear relationship between the average biomass per specimen and date. Using this general approach, we then constructed three hypothetical scenarios, which differ in how the *slope* of this linear relationship varies across years. In each scenario we only considered positive slopes, following the well-established general positive relationship between animal body size and development time [62]. In Scenario I we assumed that this slope was identical for all five years of simulated data (i.e. there is no directional change in the relationship between the average biomass per specimen and date across years). In Scenario II we assumed that the slope became progressively steeper across years. This scenario reflects the findings by [48], in which warming resulted in an advancing phenology and smaller body size of tephritid flies early in the season, and a delayed phenology and larger body size of these flies later in the season. In Scenario III we assumed that the slope became less steep across years, which reflects the empirical situation as observed in phytoplankton [44], in which smaller-sized dinoflagellates advanced their phenology, resulting in increased overlap of their phenological distribution with that of larger-sized diatoms. For each scenario we then estimated the annual peak of the biomass distribution by calculating the date at which 50% of cumulative biomass was reached. This cumulative threshold metric of phenology is consistent with the approached used for the sanderling case above, and is frequently used in other studies [33,39,63].

## 3. Results

### (a) Estimates of phenological synchrony are reversed between arthropod biomass and arthropod abundance

**Figure 1.**
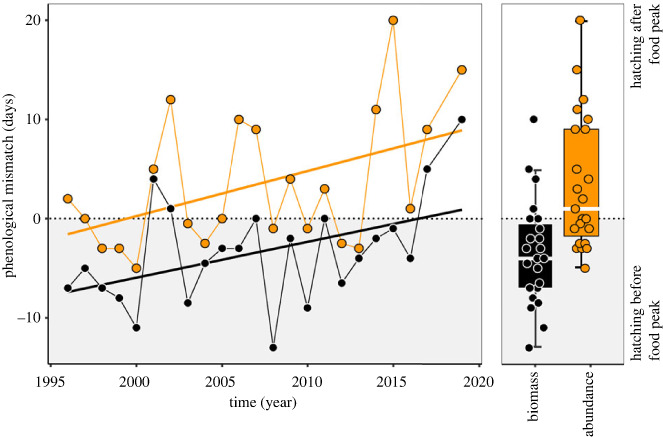
Mismatch between sanderling median hatch dates and the date when 50% of cumulative arthropod abundance (orange) or cumulative arthropod biomass (black) was sampled in pitfalls in Zackenberg (1996–2019, excluding 2018). Positive values indicate that the median hatch date occurred after the 50% date in arthropod abundance or biomass. Fitted linear models are shown as solid straight lines. Boxplots summarize the spread in the data, where horizontal white bars indicate the median, the box depicts the interquartile range and whiskers represent 1.5 times the interquartile range from the upper/lower quartile. For visual clarity we applied a horizontal jitter to the raw data depicted in the boxplots.

Our perception of how sanderling breeding was timed with respect to arthropod phenology was fully determined by our choice of currency. Overall, the calculated median date of cumulative arthropod biomass occurred on average 6.9 [95% CI: 5.1, 8.9] days after the median date of cumulative arthropod abundance, but this difference could be as large as 21 days (year 2015; figure 1). When characterizing prey phenology using arthropod abundance, the median hatch date of sanderlings occurred on average 3.5 [95% CI: 0.9, 6.3] days *after* the median date of cumulative arthropod abundance. By contrast, when characterizing prey phenology using arthropod biomass, the median hatch date of sanderlings occurred on average 3.4 [95% CI: 1.2, 5.4] days *before* the median date of cumulative arthropod biomass. Given this difference in relative timing, sanderling hatch dates became increasingly *asynchronized* over time with the median date of cumulative arthropod abundance and increasingly *synchronized* with the median date of cumulative arthropod biomass (figure 1). However, if both trends would continue linearly, sanderling hatch dates would eventually become increasingly asynchronized with both arthropod abundance and biomass. The rate of phenological change (i.e. slope) did not differ depending on the currency used to describe arthropod phenology, as for arthropod abundance the rate of change was 0.5 [95% CI: 0.1, 0.8] days per year, while for arthropod biomass the rate of change was 0.4 [95% CI: 0.0, 0.6] days per year.

**Figure 2.**
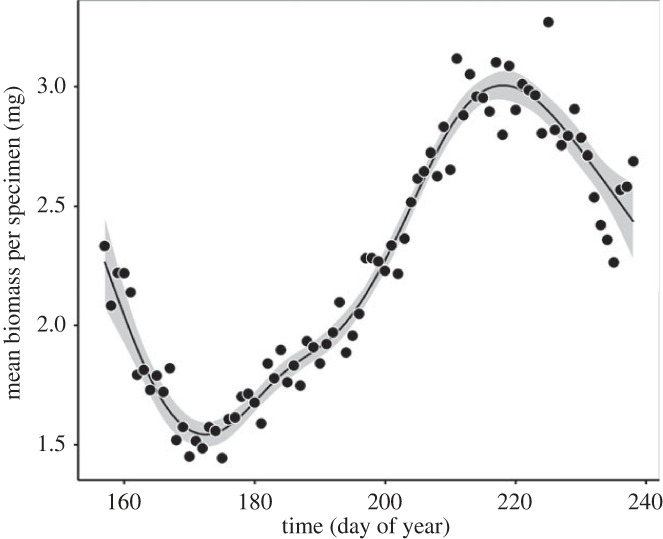
Average body mass per arthropod specimen per day at Zackenberg averaged over 23 years from 1996 to 2019 (excluding 2018). Black dots indicate the mean biomass per specimen per day averaged over all years. Only arthropod specimens of the orders Araneae, Coleoptera, Diptera, Hemiptera, Hymenoptera and Lepidoptera are included, as forming the main part of sanderling diet. Day of year 160 corresponds to 8–9 June.

Averaged across all years and taxonomic groups, mean body mass per specimen at Zackenberg increased over the season, although this relationship was nonlinear (figure 2) and varied substantially between years (electronic supplementary material, appendix S1 and figure S1).

### (b) Rates of phenological change over years depend on the seasonal pattern of biomass

**Figure 3.**
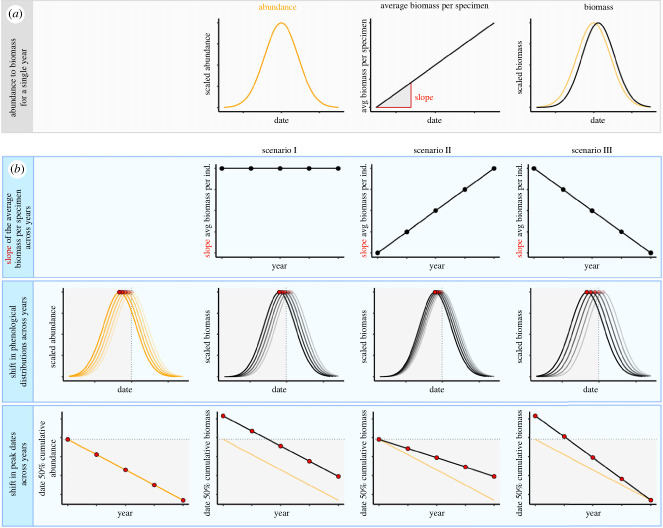
Conceptual illustration of how the use of abundance or biomass as currency for calculating resource phenology can result in different estimates for rates of phenological shifts, depending on the relationship of the average biomass per individual with date. (*a*) The phenological distribution of biomass (black) occurs after that of abundance (orange) when there is an increase in the average biomass of an individual with date. (*b*) Interannual changes in the phenological distributions of abundance and biomass. In all scenarios the phenological distribution of abundance (orange lines) and its peak date (red dots) advances linearly. Five different years are depicted as lines of varying transparency where the first year is the most transparent and the last year the least transparent. The corresponding shift in the phenological distributions (black lines) and peak (red dots) of biomass depends on the seasonal size distribution of the community across years. We assess three hypothetical scenarios: In Scenario I, the increase in the average biomass of an individual with date is consistent across years. In Scenario II, the increase in average individual biomass with date becomes steeper across years, while in Scenario III, the increase in average individual biomass with date levels off across years.

Our conceptual simulation illustrated that the peak in biomass occurred later than the peak in abundance when the average biomass per specimen increased with date (figure 3*a*). Moreover, this difference in peak dates between abundance and biomass was larger when considering a right-skewed abundance distribution, than when considering a symmetrical abundance distribution (electronic supplementary material, appendix S1 and figure S2). Our conceptual simulation illustrated that rates of phenological change for abundance and biomass differed depending on the considered scenario: In Scenario I, the increase in the average biomass per specimen with date (i.e. slope) was consistent among years, which resulted in a consistently later peak (of the same magnitude) in biomass as compared to abundance. Consequently, the rate of phenological change in biomass was identical to the rate of phenological change in abundance (figure 3*b*). In Scenario II, the increase in average biomass per specimen with date became progressively steeper across years, which caused the phenological advance of the abundance distribution to be almost completely offset by the increasingly later phenology of biomass as compared to abundance. The latter resulted in a slower rate of advancement of the peak in biomass as compared to the peak in abundance (figure 3*b*). In Scenario III, the increase in average individual biomass with date became less steep across years, resulting in a gradually increasing difference between the phenological distributions in abundance and biomass. As a result, the peak in biomass advanced faster than the peak in abundance (figure 3*b*).

## 4. Discussion

Whether the asynchrony between consumer and resource increases or decreases is an important criterion for determining trophic mismatches [9]. Examining a 23-year time series of insectivorous birds and their prey, we found that differences in phenological patterns of arthropod biomass and arthropod abundance are large enough to completely reverse the interpretation of this criterion (i.e. whether the synchrony between birds' hatch dates and the phenology of their arthropod prey increases or decreases over time). Moreover, using a conceptual simulation we indicate that rates of phenological change of abundance and biomass can differ substantially depending on the relationship of the average biomass per individual with date. Our results thus have important biological implications for the understanding of trophic mismatches in consumer–resource interactions. Importantly, our definitions of synchrony merely include the extent to which the phenology of the consumer aligns with that of the resource, without qualifying or implying any associated fitness consequences, as the latter requires knowledge on age- and temperature-dependent energy intake and energy expenditure [16].

As outlined in our conceptual simulation, differences between estimates of prey phenology based on abundance or biomass will ultimately be caused by seasonal patterns in the average body mass of prey. Indeed, we found that, averaged across years and taxonomic groups, mean body mass per arthropod specimen increased over the season in our study site, although this relationship was nonlinear and varied between years. Seasonal trends in the average body mass per arthropod specimen have also been observed by Tulp & Schekkerman [47], who attributed this pattern to phenological differences between arthropod families. In our analysis, the observed seasonal increase in mean body mass per specimen can only be explained by a later emergence of taxa with heavier specimens, because we allocated lengths to all specimens in our dataset by random sampling from a taxon- and life-stage-specific length distribution. This length-sampling procedure thus excludes seasonal trends in the average biomass per specimen within taxa. This is, however, justified at the level of individual specimens, as growth in insects generally ceases after the larval or nymphal stage [64].

The relationship between the average biomass per specimen and date is not the only component affecting the degree to which phenological estimates of abundance and biomass differ. This also depends on the shape of the phenological distribution of abundance. Our conceptual simulation illustrated that the phenological difference between abundance and biomass was larger when the phenological distribution of abundance was skewed instead of symmetrical. This conclusion, however, relies on the assumption that there was a linear increase in average biomass per specimen with date in our simulation. More generally, therefore, phenological differences between both currencies will be largest when there is a pronounced peak in abundance that corresponds to either relatively small or large specimens. In arthropods, this may for instance be caused by the highly synchronous emergence of small chironomid flies [51,65], as smaller individuals are in general also more abundant [66]. This implies that the relationship between the average biomass per specimen and date will have a more limited effect on phenological estimates for both currencies in years without pronounced peaks in prey abundance.

Our results did not reveal any detectable impact of using prey abundance or biomass on the rates of phenological change across the study period (i.e. we only found a difference in phenological intercepts, not in their slopes). However, we did find large interannual variation in the phenological differences between both currencies among years (up to 21 days in 2015). The magnitude of this difference could potentially allow for different rates of phenological change if there would have been directional trends across years (i.e. the difference between phenological estimates for abundance and biomass would have increased or decreased over time). Our conceptual simulation demonstrates that rates of phenological change based on biomass and abundance are expected to be equivalent when the relationship between the average biomass per specimen and date shows no directional change across years. By contrast, if this relationship shifts across years, the estimated rate of phenological change based on abundance or biomass will differ. The three considered scenarios in our simulation are qualitatively based on empirical studies in which there are size-specific differences in phenological change among resource taxa [44,48]. However, seasonal trends in the size distributions of prey communities can also shift due to changes in the relative abundance of individuals of different sizes across years [67,68], or due to changes in the size of individual specimens or cells [68,69]. The occurrence of these changes in prey communities emphasizes the key relevance of the choice of currencies in studies of trophic mismatches.

We show that biological inferences regarding trophic mismatches are conditional on the currency used to calculate prey phenology. This leads to the question of which currency to favour. Methodological choices should be driven by the biological question addressed [31,33,34] and can be based on mechanistic knowledge of a system such as the life histories of interacting species [27] or the type of interaction. For instance, from a consumer's perspective, the biomass of the resource is generally a more important currency than its abundance [11,46], since the former provides more ecologically relevant information regarding energetic contents. Nevertheless, intake rates of consumers might be more related to prey abundance when large prey occur patchily and numerous small prey occur more homogeneously [70]. In other systems, seasonal trends in nutritional quality might be a more important measure than either the abundance or biomass of the resource [12,71,72]. Our study demonstrates that different currencies may result in contradictory conclusions about trophic mismatches. While biomass-based estimates may often provide the most relevant metric for trophic mismatches, given the energy limitation on growing offspring [73,74], the choice of an appropriate currency would depend on mechanistic knowledge of the study system. However, study- and system-specific selection of currencies may complicate the use of a common methodology required for large-scale comparative analyses [16]. We hope that our study contributes towards the development of a solid basis for global syntheses of how consumer–resource interactions are changing with climate change.

## Data Availability

Part of the data used for this research is publicly available [50]. All other data and novel code have been made available in a permanent Dryad repository [61]. Supplementary material is available online [75].
